# Effects of Red Ginseng Extract on the Pharmacokinetics and Elimination of Methotrexate via Mrp2 Regulation

**DOI:** 10.3390/molecules23112948

**Published:** 2018-11-12

**Authors:** Sowon Lee, Mihwa Kwon, Min-Koo Choi, Im-Sook Song

**Affiliations:** 1 College of Pharmacy and Research Institute of Pharmaceutical Sciences, Kyungpook National University, Daegu 41566, Korea; okjin917@hanmail.net (S.L.); mihwa_k@naver.com (M.K.); 2 College of Pharmacy, Dankook University, Cheon-an 31116, Korea

**Keywords:** red ginseng extract (RGE), multidrug resistance-associated protein 2 (Mrp2), pharmacokinetics, methotrexate

## Abstract

We aimed to investigate the effects of red ginseng extract (RGE) on the expression of efflux transporters and to study the pharmacokinetics of representative substrate. For this, rats received single or repeated administration of RGE (1.5 g/kg/day) for 1 and 2 weeks via oral gavage. mRNA and protein levels of multidrug resistance-associated protein2 (Mrp2), bile salt export pump (Bsep), and P-glycoprotein (P-gp) in the rat liver were measured via real-time polymerase chain reaction and Western blot analysis. Ginsenosides concentrations from the rat plasma were also monitored using a liquid chromatography–tandem mass spectrometry (LC–MS/MS) system. Plasma concentrations of ginsenoside Rb1, Rb2, Rc, and Rd following repeated administration of RGE for 1 and 2 weeks were comparable but significantly higher than those after single administration of RGE. These dosing regimens did not induce significant biochemical abnormalities in the liver, kidneys, and lipid homeostasis. In the RGE repeated oral administration groups, the mRNA and protein levels of Mrp2 significantly decreased. Accordingly, we investigated the changes in the pharmacokinetics of methotrexate, a probe substrate for Mrp2, following intravenous administration of 3 mg/kg methotrexate to rats in the RGE 1-week repeated oral administration group, compared to that in the control group. Biliary excretion, but not urinary excretion, of methotrexate decreased in the RGE repeated administration group, compared to that in the control group. Consequently, the plasma concentrations of methotrexate slightly increased in the RGE repeated administration group. In conclusion, repeated administration of RGE for 1 week resulted in a decrease in Mrp2 expression without inducing significant liver or kidney damage. Pharmacokinetic herb–drug interaction between RGE and methotrexate might occur owing to the decrease in the mRNA and protein levels of Mrp2.

## 1. Introduction

Ginseng (the roots and rhizomes of *Panax ginseng* C.A. Meyer) has been extensively used for more than 2000 years in East Asian countries [1,2]. Red ginseng extract (RGE) is produced from 6-year-old fresh ginseng by steaming and drying, which leads to biochemical transformation of various ginsenosides [3]. Ginsenosides are considered the major active pharmacological constituents of ginseng. They have been shown to exhibit anti-neoplastic, anti-hypertensive, anti-diabetic, anti-inflammatory, anti-oxidant, anti-allergic, neuroprotective, and immunological effects [4,5,6,7].

Similar to other herbal medicines, ginseng products are frequently co-administered with prescribed Western medications. Accordingly, the potential for pharmacokinetic herb–drug interactions between ginseng and concomitantly administered drugs should be evaluated since they may result in toxicity or treatment failure [8]. Ginseng could alter the activity of cytochrome P450 (CYP) enzymes, the most important drug-metabolizing enzymes, which can affect the pharmacokinetics and efficacy of the co-administered drug. Administration of *Panax ginseng* for 4 weeks (1.5 g/day) resulted in slight inhibition of CYP2D6 activity, with no significant effects on CYP3A4, CYP1A2, and CYP2E1 in elderly subjects [9]. Two-week red ginseng treatment (10 mL of concentrated red ginseng extracts, dried ginseng 64%) weakly inhibited CYP2C9, CYP2D6, and CYP 3A4 activity, whereas it weakly induced CYP2D6 activity [10].

Besides inhibition or induction of CYP, the modulation of drug-transporters is also an important mechanism for herb–drug interaction [11,12,13]. Drug efflux transporters such as multidrug resistance-associated protein (Mrp), bile salt export pump (Bsep), and P-glycoprotein (P-gp) play important roles in the efflux of their substrates from the cells, thereby protecting the cells against these high concentrations of substrates, reducing intestinal absorption, or facilitating excretion of the substrate drugs [14]. Moreover, alteration of efflux transporter activity or expression could affect the tissue distribution, systemic disposition, and intestinal absorption of substrate drugs, thereby affecting their efficacy [15,16]. Despite the growing understanding of the role of the transport system in the pharmacokinetics, drug response, and herb–drug interactions, the effects of red ginseng on the efflux transporters have not yet been fully elucidated. A clinical study reported that red ginseng exhibited limited effects on P-gp function following 2-weeks repeated administration of red ginseng product in humans [10].

Therefore, in the present study, we investigated the effects of single or repeated administration of RGE on efflux transporters and examined the potential changes in the pharmacokinetics of their substrate drugs. Of these, Mrp2 drew our interest because it is mainly involved in the pharmacokinetics and renal and biliary elimination of its substrate anions as well as amphipathic drugs that conjugate with glucuronide, sulfate, and glutathione (GSH) [17,18]. For example, numerous anionic drugs such as methotrexate, SN38, cisplatin, vinblastine, and sulfinpyrazone are mainly eliminated from the body by Mrp2 [19,20]. GSH facilitates the biliary excretion of amphipathic drugs via Mrp2 through the conjugation reaction and also acts as a regulator of redox homeostasis [21]. RGE has been shown to be effective in reducing oxidative stress through the Nrf2 signaling pathway [7,22]. Nrf2 is a transcriptional factor that regulates Mrp2 expression [21], suggesting herb–drug interaction between red ginseng and Mrp2 substrates.

## 2. Results

### 2.1. Concentration of Ginsenosides in Red Ginseng Extracts and Rat Plasma

To measure ginsenoside concentrations, we developed analytical methods for quantification of 14 ginsenosides in diluted RGE and rat plasma samples with slight modification of a previously described method [23]. Among the 14 ginsenosides tested, eight ginsenosides (Rb1, Rb2, Rc, Rd, Rg3, Re, Rh1, and Rg1) could be quantitated in the diluted RGE. Peaks of the other six ginsenosides (Rh2, F2, compound K, protopanaxadiol, F1, and protopanaxatriol) were not detected since these ginsenosides are minor components and are biological metabolites of Rb1, Rb2, Re, or Rg1 that could be produced via metabolism in the gut microflora [24,25]. The contents of ginsenosides could differ depending on the preparation procedure for RGE (steaming and drying conditions); however, the contents of major ginsenosides, such as Rb1, Rb2, Rc, and Rg3, were comparable to previously reported values [26,27]. Ginsenoside contents in RGE used in this study are summarized in Table 1. Rb1 content was the highest (0.19%), and Rb2, Rc, and Rg3 contents were 0.09%, 0.1%, and 0.13%, respectively. Rd, Re, and Rh1 contents in RGE were in the range of 0.05–0.06%.

**Figure 1:**
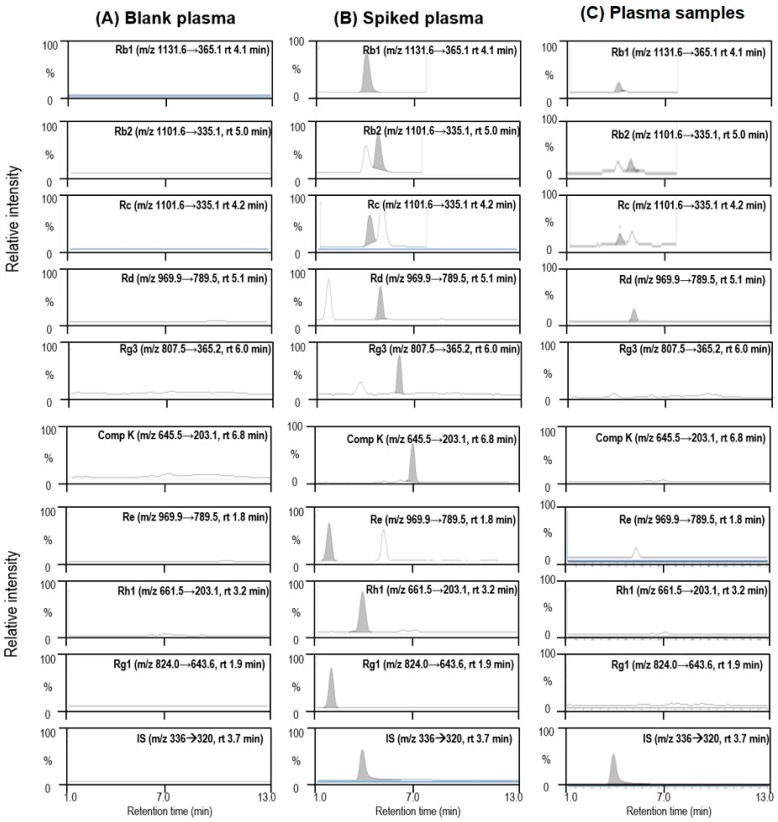
Representative multiple reaction monitoring (MRM) chromatograms of ginsenosides Rb1, Rb2, Rc, Rd, Rg3, compound K (Comp K), Re, Rh1, Rg1, and berberine (internal standard, IS) in (**A**) rat blank plasma, (**B**) standard ginsenosides (20 ng/mL) spiked in rat blank plasma, and (**C**) plasma samples following single oral administration of RGE (1.5 g/kg).

After RGE was orally administered to rats at single or multiple doses for 1 or 2 weeks, the plasma concentrations of ginsenosides were also monitored. Among the 14 ginsenosides monitored, only four ginsenosides (Rb1, Rb2, Rc, and Rd) could be quantitated (Table 2); however, the other 10 ginsenoside peaks were not detected in rat plasma. The representative multiple reaction monitoring (MRM) chromatograms for the four identified ginsenosides (Rb1, Rb2, Rc, and Rd) and five unidentified ginsenosides (Rg3, compound K, Re, Rh1, and Rg1) are shown in Figure 1. Plasma concentrations of ginsenosides Rb1, Rb2, and Rc following single oral administration (SA) of RGE were comparable to each other and greater than the plasma concentrations of Rd (Table 2). Repeated administration of RGE for 1 week (1WRA) resulted in an increase in plasma ginsenoside concentrations, compared to those in the SA group; however, they were not significantly different from those in the 2WRA group (Table 2). The results suggested that ginsenosides accumulated in the plasma because of their long elimination half-life (t_1/2_). For example, Rb1 is the most abundant and stable ginsenoside with a long elimination half-life of 58.47 h [28,29]. The results also suggested that the steady-state plasma concentrations were reached within 1 week and maintained up to 2 weeks. Although the steady-state plasma concentrations of ginsenosides did not match with the order of ginsenoside contents in RGE (Table 1 and Table 2), the major components in RGE (Rb1, Rb2, and Rc) existed in the rat plasma at the highest concentration levels. Thus, it was suggested that Rb1, Rb2, and Rc could permeate the intestinal membrane despite their hydrophilicity and large molecular size based on their multiple glycoside conjugation [3,30].

### 2.2. Analysis of the mRNA Expression of Efflux Transporters

**Figure 2:**
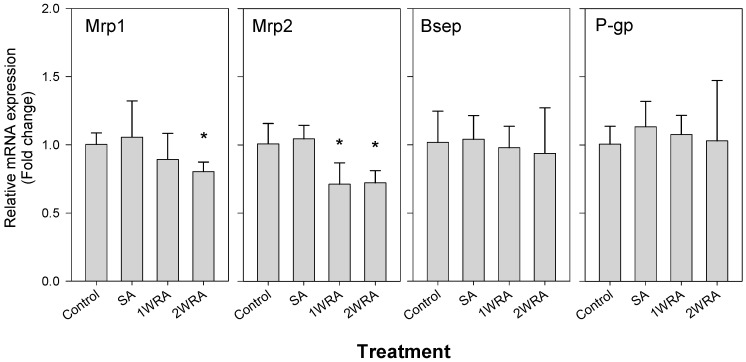
mRNA expression level of multidrug resistance-associated protein 1 (Mrp1), Mrp2, bile salt export pump (Bsep), and P-glycoprotein (P-gp) in the liver of rats in the control, single administration (SA), and repeated administration of RGE (1.5 g/kg, PO) for 7 days (1WRA) and 14 days (2WRA) groups. Hrpt1 was used as an internal standard. Each bar represents the mean ± SD from four rats per group. * *p* < 0.05, significant compared with SA group by Student’s *t*-test.

Because of the potential of drug interactions between herbal medicines and efflux transporters, we measured the mRNA expression of efflux transporters, such as Bsep, P-gp, Mrp1, and Mrp2 in the liver tissues from rats in the control, SA, 1WRA, and 2WRA groups (Figure 2). The expression of P-gp, and Bsep did not change; however, Mrp1 expression slightly decreased in both single- and multiple-dose RGE-treated groups although it did not reach statistical significance. Mrp2 expression slightly decreased in the SA group but significantly decreased in the repeated-dose RGE-treated groups. There was no significant difference in Mrp2 expression between the 1WRA and 2WRA groups. Based on the plasma ginsenoside concentrations and mRNA expression level, further comparison studies were performed among the control, SA, and 1WRA groups.

### 2.3. Western Blot Analysis of Mrp2

**Figure 3:**
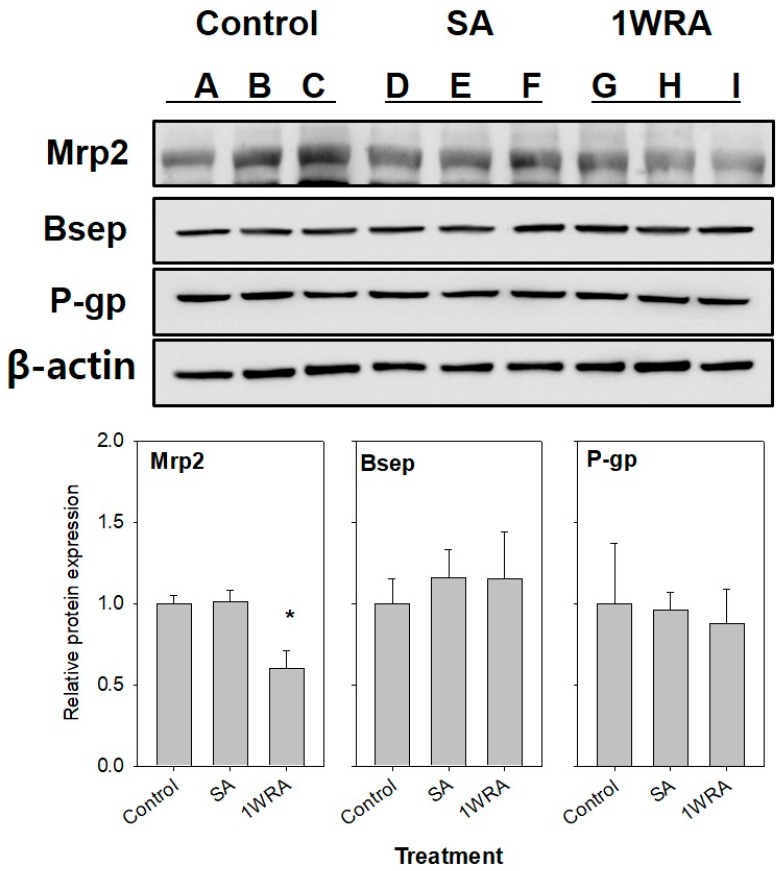
Protein expression of Mrp2 in the liver of rats in the control (lanes A, B, and C), SA (lanes D, E, and F), and 1WRA (lanes G, H, and I) groups. β-actin served as a loading control. Quantitative analysis of Western blot results is shown in the lower panel. Each bar represents the mean ± SD of three independent densitometric analyses. * *p* < 0.05, significant compared with SA group by Student’s *t*-test.

To confirm the decrease in the mRNA expression of Mrp2 in the repeated-dose RGE-treated groups, Western blot analysis was performed using the liver tissues collected from rats in the control, SA, and 1WRA groups. As shown in Figure 3, Mrp2 protein levels significantly decreased in the liver tissues of rats in the 1WRA group, compared to that in the control group.

### 2.4. Effect of RGE Treatment on Biochemical Parameters for Liver and Renal Function 

The levels of alanine aminotransferase (ALT) and aspartate aminotransferase (AST) were not significantly affected by multiple-dose administration of RGE (1.5 g/kg/day), suggesting that it had negligible effects on liver function (Table 3). Additionally, no changes were observed in the levels of triglycerides, total cholesterol, high-density lipoprotein (HDL) cholesterol, low-density lipoprotein (LDL) cholesterol, and free fatty acids in the multiple-dose RGE (1.5 g/kg/day)-treated group, indicating that RGE had negligible effects on lipid homeostasis (Table 3).

### 2.5. Pharmacokinetics of Methotrexate

Next, we investigated the effects of RGE on the biliary excretion of Mrp2 substrate drug in rats. Methotrexate was selected as a representative substrate for Mrp2 because 62% and 27% of methotrexate intravenous dose is excreted into the bile and urine, respectively, within 3 h mainly by Mrp2 [31].

**Figure 4:**
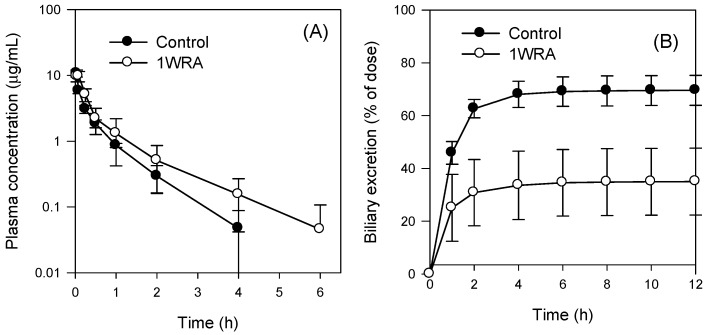

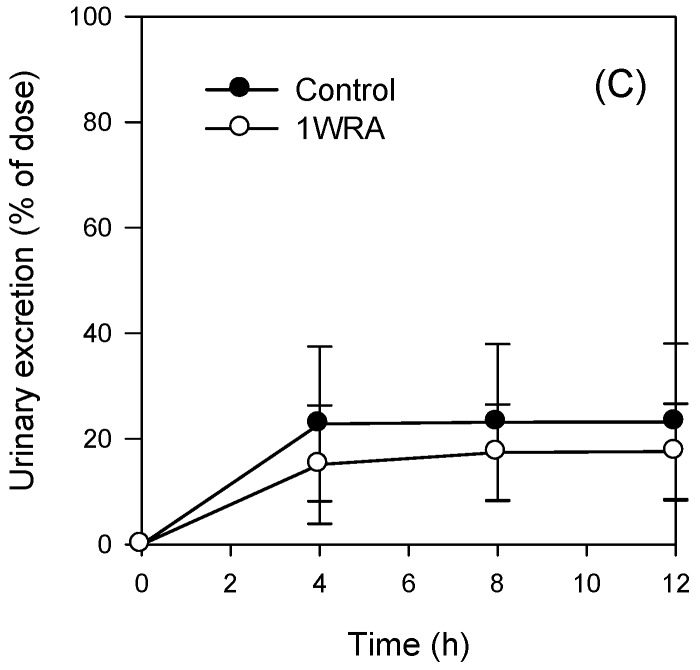
(**A**) Plasma concentration-time profile, (**B**) biliary excretion, and (**C**) urinary excretion of methotrexate following intravenous injection of methotrexate at a dose of 3 mg/kg in rats from the control and 1WRA groups of RGE (1.5 g/kg, PO). Data represent the means ± SD (*n* = 3 or 4 rats/group).

Pharmacokinetic parameters of methotrexate related to its excretion were presented in Figure 4 and Table 4. We observed delayed disposition of methotrexate from the plasma (Figure 4), including an increase in t_1/2_, in the 1WRA group, which resulted in a significant decrease in the CL_total_ and a significant increase in the area under plasma concentration-time curve (AUC) of methotrexate (Table 4).

Since methotrexate underwent the least metabolism [31], disposition from the plasma could be attributed to its excretion via the liver and kidney. Multiple-dose administration of red ginseng decreased the biliary excretion, but not the urinary excretion, of methotrexate (Figure 4B,C) and consequently, CL_bile_ of methotrexate decreased; however, its CL_urine_ did not change in the 1WRA group (Table 4). CL_bile_ accounted for 70% of the CL_total_ of the intravenous bolus injection of methotrexate at a dose of 3 mg/kg in the control group and CL_urine_ accounted for a relatively minor portion (18%, Table 4) in this group. These results showed that biliary excretion is the major pathway of methotrexate elimination, which is consistent with the findings of a previous study [21] and Mrp2 could be a controlling factor in the pharmacokinetics of methotrexate [18]. Therefore, the decrease in the expression of Mrp2 might contribute to the decreased biliary excretion of methotrexate and consequently a 1.5-fold increase in plasma methotrexate exposure (Table 4).

## 3. Discussion

RGE treatment at a daily dose of 1.5 g/kg for 1 week resulted in steady-state plasma concentrations of major ginsenosides, such as Rb1, Rb2, Rc, and Rd (Table 2). In addition, this treatment did not induce biochemical abnormalities in the liver, kidneys, and lipid homeostasis in rats (Table 3). The plasma concentration of Rb1 was the highest among the ginsenosides detected in rat plasma after oral administration of RGE, followed by Rb2, Rc, and Rd, which was similar to the rank of the ginsenoside content in RGE used in this study (Table 1 and Table 2). However, other ginsenosides, such as Rg3, Re, and Rh1, were not detected in rat plasma despite their high content in RGE (Table 1 and Table 2). Although the underlying mechanisms need to be explored, limited intestinal permeability, short plasma half-life, and intestinal instability owing to the biotransformation and pH-dependent degradation of Rg3, Re, and Rh1 [32,33,34], compared to Rb1, Rb2, Rc, and Rd, might contribute to this finding.

Repeated RGE treatment decreased the mRNA and protein expression of Mrp2; however, it did not modulate the expression of Bsep and P-gp in the liver (Figure 2 and Figure 3). Nuclear receptors, such as farnesoid X receptor (FXR, also known as bile acid receptor) and pregnane X receptor (PXR), are known as major regulators of the expression of Bsep and P-gp, respectively [35]. The expression of Mrp2 has been shown to be regulated through transcriptional and post-transcriptional regulation via activation of the Nrf2 and cAMP-dependent protein kinase A (PKA) signaling pathways [5]. Repeated administration of RGE reduced Mrp2 expression; however, the expression of Bsep and P-gp did not change, suggesting that multiple-dose RGE treatment might affect the Nrf2 pathway. This might subsequently contribute to the decrease in Mrp2 mRNA and protein expression, and the increase in the plasma concentration of MTX, an Mrp2 probe substrate drug, by decreasing its elimination and biliary excretion following repeated administration of RGE (Figure 4). Therefore, it could be concluded that multiple-dose administration of RGE could induce herb–drug interactions with various Mrp2 substrate drugs by reducing the hepatobiliary elimination of the Mrp2 substrates, xenobiotics, and their conjugated metabolites, which should be considered when Mrp2 substrate drugs are co-administered with RGE.

In addition, MTX has been used as a chemotherapeutic and immunosuppressive agent for treatment of leukemia, lymphoma, rheumatoid arthritis, and Crohn’s disease [24]. In particular, the beneficial effects of red ginseng products for reducing the symptoms of rheumatoid arthritis have been proven in animals and humans [24,25,26,27,36]. Therefore, the use of MTX and red ginseng alone or in combination is highly plausible for treatment of rheumatoid arthritis. Repeated high doses of red ginseng could decrease Mrp2 expression, which is crucial for the elimination of MTX. Therefore, in conclusion, this study showed that the herb–drug interactions between red ginseng and MTX occurred via Mrp2 regulation changes in rats, but clinical significance thereof should be determined with follow-up studies.

## 4. Materials and Methods

### 4.1. Materials

Korean red ginseng extract (RGE) was obtained from Punggi Ginseng Cooperative Association (Punggi, Korea). Methotrexate was purchased from Sigma-Aldrich (St. Louis, MO, USA). Analytical standard ginsenosides were purchased from Ambo Institute (Daejeon, Korea).

### 4.2. Animals

Male Sprague−Dawley rats (7–8 weeks, 220−250 g) were purchased from Samtako Co. (Osan, Korea). All animal procedures were approved by the Animal Care and Use Committee of Kyungpook National University (Approval No. 2017-0021) and carried out in accordance with the National Institutes of Health guidance for the care and the use of laboratory animals.

### 4.3. RGE Administration

Rats in the control group received water (2 mL/kg as a vehicle treatment) orally at 9 a.m. for 7 days by oral gavage. RGE suspension (1.5 g/kg/day, 2 mL/kg suspended in water) was administered to rats by oral gavage at 9 a.m. once (SA group), for 7 days (1WRA group), and 14 days (2WRA group), respectively. Two hours after the last treatment of RGE, abdominal arterial blood (about 5 mL) and liver tissues were collected from rats in all groups. The liver tissues were snap-frozen for analysis of the mRNA and protein expression of efflux transporters. The plasma samples were used for measurement of plasma ginsenoside concentrations and biochemical parameters. The biochemical parameters such as ALT, AST, triglycerides, plasma cholesterol levels, free fatty acids, blood urea nitrogen and serum creatinine were measured from the service of Seoul Clinical Laboratories (Yongin, Korea) using UV spectrophotometric assay kits from Young-Dong Diagnostics Co. (Yongin, Korea). Lyphocheck assayed chemistry control (normal and abnormal standards; Bio-Rad, Hercules, CA, USA) were used as positive control and YD calibrator (Young-Dong Diagnostics Co.) was used for external calibration.

### 4.4. Bioanalysis of 14 Ginsenosides

Ginsenoside concentrations in RGE and plasma samples were analyzed using an Agilent 6470 triple quadrupole liquid chromatography–tandem mass spectrometry (LC–MS/MS) system (Agilent, Wilmington, DE, USA) equipped with an Agilent 1260 high-performance liquid chromatography (HPLC) system according to the previously published method [23]. Briefly, diluted RGE samples (50 μL) and plasma samples (50 μL) were protein-precipitated with methanol 200 μL containing berberine (0.5 ng/mL; used as an internal standard). Aliquots (10 μL) of the supernatant were injected into the LC–MS/MS system for the analysis of ginsenosides. Ginsenosides were separated on a Synergi Polar RP column (2 × 150 mm, 4 μm particle size; Phenomenex) using a mobile phase consisting of water (A) and methanol (B) containing 0.1% formic acid at a flow rate of 0.3 mL/min. The solvent gradient program was as follows: (1) 0–1 min, 70 % B; (2) 1–6.5 min, 90 % B; (3) 6.5–7 min, 80% B; and (4) 7–14 min, 70% B.

Quantification of a separated ginsenoside peak was performed using MRM mode at *m*/*z* 1131.6 → 365.1 for Rb1 (retention time (rt) = 4.1 min), *m*/*z* 1101.6 → 335.1 for Rb2 (rt = 5.0 min) and Rc (rt = 4.2 min), *m*/*z* 969.9 → 789.5 for Rd (rt = 5.1 min) and Re (rt = 1.8 min), *m*/*z* 823.5 → 365.1 for Rf (rt = 2.7 min), *m*/*z* 824 → 643.6 for Rg1 (T_R_ = 1.9 min), *m*/*z* 807.5 → 365.1 for Rg3 (T_R_ = 6.0 min), *m*/*z* 661.5 → 203.1 for Rh1 (rt = 3.2 min), *m*/*z* 645.5 → 645.5 for Rh2 (rt = 6.9 min), *m*/*z* 661.5 → 203.1 for F1 (rt = 3.7 min), *m*/*z* 807.5 → 627.5 for F2 (rt = 6.1 min), *m*/*z* 645.5 → 203.1 for compound K (rt = 6.8 min), *m*/*z* 483.4 → 483.4 for protopanaxadiol (rt = 6.8 min), *m*/*z* 499.4 → 499.4 for protopanaxatriol (rt = 6.8 min), and *m*/*z* 336.1 → 320 for berberine (internal standard, IS) (rt = 3.7 min) in the positive ionization mode with collision energy (CE) of 30–65 eV.

### 4.5. Real-Time Reverse-Transcription Polymerase Chain Reaction (RT-PCR) Analysis

Total RNA was extracted from liver samples (100 mg) using Qiazol (Qiagen, Valencia, CA, USA), according to the vendor’s protocol. The concentration of total RNA was determined by Nano Vue Plus (GE healthcare Korea, Seoul, Korea).

RT-PCR of Mrp1, Mrp2, Bsep, and P-gp was performed using a LightCycler 96 real-time PCR system (Roche, Carlsbad, CA, USA) as previously described [21,37]. Primers were designed using ProbeFinder as follows: 5′-tgagggtggagaaaaggttg-5′ and 5′-aaacccagggtgagagatga-3′ for Mrp1 (NM_022281.2); 5′-aatacatgaccttttggtgtttctg-5′ and 5′-acgaaaccgatcagcaactt-3′ for Mrp2 (NM_012833.2); 5′-gggcagtcacacccatctac-5′ and 5′-ctttatcgaggagtgaaaaagtcc-3′ for Bsep (NM_031760.1); 5′-cacagaccgtcagcgaca-5′ and 5′-caatgcccgtgtaatagtaggc-3′ for P-gp (NM_012623.2).

### 4.6. Western Blot Analysis

Protein expression of Mrp2, Bsep, and P-gp was measured as previously described [21]. Briefly, total protein was obtained by homogenizing 100 mg of liver samples with equal volume of a tissue lysis buffer. Protein samples (30–50 µg) were loaded and separated by sodium dodecyl sulfate–polyacrylamide gel electrophoresis (SDS-PAGE) on a 4–15% gradient gel (Bio-Rad). Separated proteins were transferred onto a Potran 0.45 μm nitrocellulose membrane (GE Healthcare). The membrane was blocked with 5% bovine serum albumin in Tris-buffered saline containing 0.1% Tween20 (TBST) (Biosesang, Seoul, Republic of Korea) for 1 h and incubated with primary antibody at 4 °C for 12 h. Primary antibodies were used as follows: anti-Mrp2 antibody (ab203397, dilution 1:1000, Abcam, San Francisco, CA, USA), anti-Bsep antibody (ab217532, dilution 1:1000, Abcam), anti-P-gp antibody (E1Y7S, dilution 1:1000, Cell signaling technology, Danvers, MA, USA) and beta-actin antibody (1:1000, Cell signaling technology). The membrane was rinsed twice with TBST at 25 °C and treated with horseradish peroxidase-labeled anti-rabbit IgG antibody (Santa Cruz Biotechnology, Dallas, TX, USA). Protein bands were visualized using a Luminata Forte enhanced chemiluminescence system (Millipore, Burlington, MA, USA), and beta-actin served as the loading control.

### 4.7. Pharmacokinetics of Methotrexate

Pharmacokinetic study of methotrexate was performed as previously described [21]. Briefly, the femoral arteries, femoral veins, and bile duct of rats in the control and 1WRA groups were cannulated with PE50 or PE10 polyethylene tubing (Jungdo, Seoul, Korea) under anesthesia. Pharmacokinetic studies started 2 h after the last RGE treatment.

Methotrexate solution (3 mg/kg in phosphate-buffered saline) was injected intravenously to rats. Blood samples (about 150 μL) were collected from the femoral artery at 0, 0.03, 0.08, 0.25, 0.5, 1, 2, 4, and 6 h after the methotrexate injection. Bile samples were collected at 1 and 2 h and every 2 h up to 12 h through the bile cannula. Urine samples were also collected every 4 h up to 12 h through urinary bladder.

Aliquots (50 μL) of plasma, bile, and urine samples were added to 250 μL of acetonitrile containing 2 ng/mL of propranolol (IS). After vortexing for 10 min and centrifugation at 10,000× *g* for 10 min, an aliquot (1 μL) of the supernatant was injected directly into the Agilent 6430 triple quadrupole LC–MS/MS system, according to the previously described method [21]. Briefly, methotrexate and propranolol (IS) were eluted at 2.1 and 2.7 min, respectively, on a Synergi Polar RP column. Mobile phase consisted of water (0.1% formic acid):acetonitrile (0.1% formic acid) = 20:80 (*v*/*v*) and eluted at a rate of 0.25 mL/min. Quantitation was carried out at *m*/*z* 455.2 → 308.1 for methotrexate and *m*/*z* 260 → 116 for IS in the positive ionization mode.

### 4.8. Data Analysis

Pharmacokinetic parameters were calculated by non-compartmental analysis using WinNonlin version 2.0 software (Pharsight, Certara, NJ, USA).

Statistical comparisons were performed by *t*-test using the Statistical Package for the Social Sciences (SPSS Inc., Chicago, IL, USA). A *p* value < 0.05 was considered statistically significant.

## Figures and Tables

**Table 1:** Contents of ginsenosides in RGE.

Ginsenoside		(μg Ginsenoside/g RGE)
20(s)-protopanaxadiol	Rb1	1925 ± 38.3
	Rb2	929.6 ± 32.0
	Rc	1015 ± 32.4
	Rd	541.6 ± 47.0
	Rh2	ND
	Rg3	1291 ± 47.5
	F2	ND
	Compound K	ND
	Protopanaxadiol	ND
20(s)-protopanaxatriol	Re	532.1 ± 36.3
	Rh1	654.2 ± 31.2
	Rg1	160.0 ± 0.4
	F1	ND
	Protopanaxatriol	ND

Data are expressed as mean ± SD from triplicated measurements of ginsenosides in RGE. ND: Not detected. RGE: Red ginseng extract.

**Table 2:** Plasma concentration of ginsenosides in rat plasma 2 h after the last oral administration of RGE at a dose of 1.5 g/kg/day.

Ginsenoside		Plasma Concentration (ng/mL)		
		SA (1.5 g/kg/day)	1WRA (1.5 g/kg/day for 1 week)	2WRA (1.5 g/kg/day for 2 weeks)
20(s)-protopanaxadiol	Rb1	8.9 ± 2.3	33.6 ± 10.1 *	32.1 ± 12.4 *
	Rb2	7.2 ± 1.6	25.1 ± 6.4 *	22.3 ± 7.8 *
	Rc	6.1 ± 1.6	25.5 ± 7.9 *	25.1 ± 8.5 *
	Rd	1.8 ± 0.6	9.8 ± 2.5 *	11.5 ± 4.5 *
	Rh2	ND	ND	ND
	Rg3	ND	ND	ND
	F2	ND	ND	ND
	Compound K	ND	ND	ND
	Protopanaxadiol	ND	ND	ND
20(s)-protopanaxatriol	Re	ND	ND	ND
	Rh1	ND	ND	ND
	Rg1	ND	ND	ND
	F1	ND	ND	ND
	Protopanaxatriol	ND	ND	ND

Data are expressed as mean ± SD from four rats of control, SA, 1WRA, and 2WRA groups. * *p* < 0.05, significant compared with SA group by Student’s *t*-test. ND: Not detected. RGE: red ginseng extract.

**Table 3:** Biochemical parameters from rat plasma following multiple administration of RGE for 1 week at a dose of 1.5 g/kg/day.

Biochemical Parameters		Control	1WRA
Alanine aminotransferase (ALT)	Unit/L	72.7 ± 13.3	66.3 ± 28.0
Aspartate aminotransferase (AST)	Unit/L	27.3 ± 3.1	41.0 ± 29.8
Triglyceride	mg/dL	80.3 ± 57.0	73.7 ± 10.5
Total cholesterol	mg/dL	62.7 ± 13.3	53.7 ± 9.1
HDL cholesterol	mg/dL	54.7 ± 9.9	49.0 ± 1.7
LDL cholesterol	mg/dL	9.0 ± 1.0	7.7 ± 1.2
Free Fatty acid	mg/dL	161.3 ± 73.0	166.0 ± 12.2
Blood urea nitrogen (BUN)	mg/dL	34.9 ± 7.2	33.4 ± 12.1
Creatinine	mg/dL	0.5 ± 0.1	0.5 ± 0.1

Data are expressed as mean ± SD from three rats of control and 1WRA groups. HDL: high-density lipoprotein; LDL: low-density lipoprotein.

**Table 4:** Pharmacokinetic parameters of methotrexate following intravenous injection of methotrexate at a dose of 3 mg/kg.

Parameters		Groups	
		Control (*n* = 4)	1WRA (*n* = 6)
C_0_	μg/mL	15.6 ± 2.6	9.7 ± 1.7 *
AUC_6h_	μg min/mL	3.8 ± 0.2	5.6 ± 2.2 *
AUC_∞_	μg·min/mL	3.8 ± 0.2	5.7 ± 2.3 *
t_1/2_	h	0.7 ± 0.2	0.9 ± 0.2 *
MRT	h	0.7 ± 0.2	0.9 ± 0.3
V_d_	mL/kg	518.1 ± 119.0	477.2 ± 109.3
CL_total_	mL/min/kg	13.1 ± 0.7	9.8 ± 3.1 *
CL_bile_	mL/min/kg	9.2 ± 1.0	4.3 ± 2.4 *
CL_urine_	mL/min/kg	2.3 ± 1.5	1.8 ± 1.1

Data are expressed as mean ± SD from four rats of control and six rats of 1WRA groups. * *p* < 0.05, significant compared with SA group by Student’s *t*-test. C_0_: initial plasma concentration. AUC_6h_ or AUC_∞_: Area under plasma concentration-time curve from zero to 6 h or infinity. t_1/2_: elimination half-life; Vd: volume of distribution at steady state. MRT: mean residence time; CL_total_: total CL (Dose/plasma AUC). CL_bile_: biliary CL (Excreted amount in bile/plasma AUC). CL_urine_: urinary CL (Excreted amount in urine/plasma AUC).
